# Hemolysis and Renal Outcomes After Pulsed Field Versus Thermal Ablation for Atrial Fibrillation: A Meta‐Analysis

**DOI:** 10.1111/pace.70225

**Published:** 2026-03-27

**Authors:** Cesar Erazo, Frans Serpa, Gabriel Cavalcante Lima Chagas, Andre Rivera, Esteban Arevalo, Jose Aguilera, Kamala P. Tamirisa

**Affiliations:** ^1^ Cleveland Clinic Cleveland Ohio USA; ^2^ University of Texas Southwestern Medical Center Dallas Texas USA; ^3^ Universidade Nove de Julho São Paulo Brazil; ^4^ Loma Linda University Loma Linda California USA; ^5^ Saint Luke's Mid America Heart Institute University of Missouri‐Kansas City Kansas City Missouri USA

**Keywords:** acute kidney injury, atrial fibrillation, catheter ablation, intravascular hemolysis, pulsed field ablation, thermal ablation

## Abstract

**Purpose:**

Evaluate the comparative risk of hemolysis and acute kidney injury (AKI) with pulsed field ablation (PFA) versus thermal ablation (TA) for atrial fibrillation.

**Methods:**

Databases were searched through June 2025 for comparative studies reporting hemolysis markers or renal outcomes. Random‐effects models estimated risk ratios (RRs) and mean differences (MDs).

**Results:**

Eight studies (*n* = 4307) were included. Compared with TA, PFA was not associated with a statistically significant increase in AKI (RR 2.00, 95% CI 0.5–7.9) or change in creatinine (MD 0.02 mg/dL, *p* = 0.10). PFA was associated with higher LDH (MD +72 U/L *p* < 0.001) and lower haptoglobin (MD −0.60 g/L *p *< 0.001).

**Conclusions:**

PFA may be associated with greater biochemical hemolysis than TA; however, no statistically significant difference in AKI or post‐procedural creatinine was observed in pooled analyses.

## 1 Introduction

Pulsed field ablation (PFA) is a non‐thermal ablation technique for atrial fibrillation (AF) that creates myocardial‐selective lesions by electroporation [1]. Although its safety profile is favorable compared with thermal ablation (TA) [1], biochemical evidence of intravascular hemolysis resulting in acute kidney injury (AKI) has been reported in post‐marketing and comparative studies [2]. The clinical significance of this observation remains incompletely understood; therefore, we performed a meta‐analysis comparing hemolytic and renal outcomes after PFA versus TA to better contextualize this risk in clinical practice.

## 2 Methods

**FIGURE 1:**
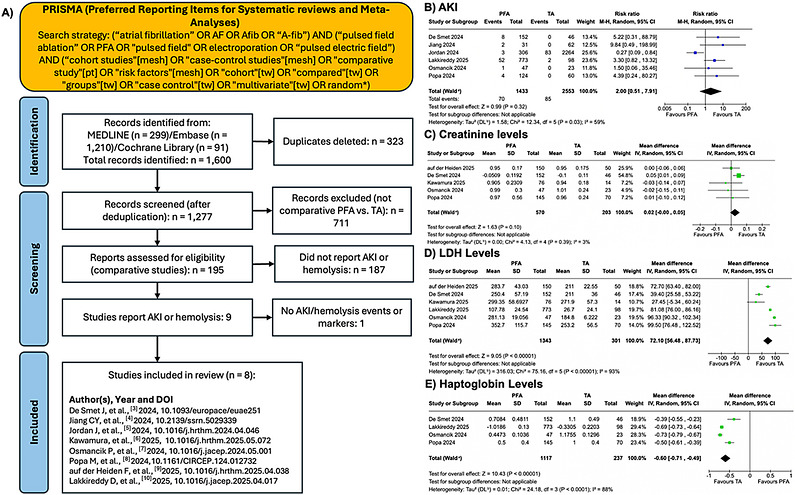
PRISMA flow and pooled analyses of renal and hemolytic outcomes comparing pulsed field ablation (PFA) with thermal ablation (TA). (A) Study selection. (B) Pooled risk ratio for AKI. (C) Mean difference in serum creatinine. (D) Mean difference in LDH. (E) Mean difference in haptoglobin. AKI, acute kidney injury; CI, confidence interval; LDH, lactate dehydrogenase; MD, mean difference; RR, risk ratio. [Colour figure can be viewed at wileyonlinelibrary.com]

We searched MEDLINE, Embase, and Cochrane Library (inception–June 2025) for comparative studies of PFA versus TA (radiofrequency [RFA] or cryoballoon ablation [CBA]) in AF that reported at least one hemolysis marker (e.g., LDH, haptoglobin) or a renal outcome (change in serum creatinine and AKI). AKI was defined using KDIGO criteria in certain studies, whereas others reported AKI without specifying diagnostic thresholds. The PRISMA flow is shown in Figure 1A. Two reviewers screened and extracted data independently; disagreements were resolved by consensus.

From each study, we captured design, baseline characteristics, ablation system/catheter, total application, and outcomes. Random‐effects models generated risk ratios (RRs) for dichotomous outcomes and mean differences (MDs) for continuous outcomes with 95% confidence intervals (CIs). Heterogeneity was quantified with I^2^. Analyses were performed in RevMan v5.4 and R v4.5.0 (*meta* package).

## 3 Results

### 3.1 Study Selection

The search identified 1600 records; after removing 323 duplicates, 1277 titles and abstracts were screened. We excluded 711 non‐comparative reports and 187 studies without AKI/hemolysis data; one additional study reported no events or markers. Eight studies met the inclusion criteria (*n* = 4307; PFA *n* = 1680; TA *n* = 2627).

### 3.2 Baseline and Procedural Characteristics

The mean age of the participants included ranged from 61 to 70 ± 10 years, with variable proportions of women across studies. When reported, mean CHA_2_DS_2_‐VASc scores ranged from 1.6 to 3.3, and mean LVEF from 54 to 66 ± 7%. PFA applications ranged from 31 ± 3 to 75 ± 29 per case across studies.

### 3.3 Meta‐Analysis Outcomes

Among the included studies, PFA was not associated with an increased risk of AKI compared with TA (RR 2.00, 95% CI 0.5‐7.9; *p* = 0.32; I^2^ = 59%), and there was no significant difference in post‐procedural creatinine levels between techniques (MD 0.02 mg/dL, 95% CI 0‐0.05; *p* = 0.10; I^2^ = 3%). PFA was associated with greater biochemical evidence of hemolysis, as indicated by higher LDH (MD +72.10 U/L, 95% CI 56.5–87.7; *p *< 0.001; I^2^ = 93%) and lower haptoglobin (MD –0.60 g/L, 95% CI −0.7 to −0.5; *p* < 0.001; I^2^ = 88%) (Figure 1 B–E).

## 4 Discussion

Our findings suggest that compared to TA, PFA was not associated with a statistically significant increase in adverse renal outcomes, including AKI or changes in serum creatinine. Despite evidence of biochemical hemolysis with PFA (higher LDH; lower haptoglobin), these changes did not seem to translate into worse renal outcomes in pooled analyses.

The hemolysis observed with PFA is most consistent with pore formation in red blood cell membranes caused by electroporation. This leads to ionic imbalance, swelling, and rupture of the cells [11]. In theory, hemolysis could contribute to AKI through tubular obstruction by heme, direct injury to kidney cells, and renal vasoconstriction [12]. However, in the included studies, creatinine changes were generally small, and clinically evident AKI events were uncommon.

Observational data suggest a potential hypothesis of a dose–response relationship between PFA pulse delivery and renal biomarker changes. In the single‐arm study by Martinez et al., pulse count was associated with creatinine rise, with an estimated increase of approximately 0.004 mg/dL per pulse [13]. This would suggest that approximately 129–140 pulses may be required to produce a clinically relevant 0.3 mg/dL increase in creatinine. In this meta‐analysis, PFA dosing was generally below these thresholds, which may potentially explain the low observed incidence of AKI despite biochemical evidence of hemolysis.

Our meta‐analysis provides a contemporary evaluation of the comparative renal outcomes of PFA versus TA. Limitations should be noted. The studies included in our analysis varied in design, patient populations, ablation protocols, outcome definitions, and timing of measurements, which may have introduced variability. Given the wide confidence intervals and moderate heterogeneity, this analysis may be underpowered for rare renal events; consequently, absence of statistical significance should not be interpreted as absence of risk.

## 5 Conclusion

This meta‐analysis suggests that the use of PFA for AF ablation may be associated with greater intravascular hemolysis compared with TA, without a significant increase in the risk of AKI. The observed pattern of intravascular hemolysis highlights the importance of identifying high‐risk patients and implementing close renal monitoring. Further research into preventive strategies and technological refinements is warranted. Large prospective studies and real‐world datasets with standardized outcome definitions are required to validate these findings and guide clinical practice.

## Author Contributions

Cesar Erazo and Gabriel Cavalcante Lima Chagas performed data extraction, analysis, and manuscript writing. Frans Serpa and Andre Rivera assisted with study conceptualization, methodology, and editing. Esteban Arevalo and Jose Aguilera contributed to data validation. Kamala P. Tamirisa supervised the research project and reviewed the final manuscript.

## Funding

This research did not receive any specific grant from funding agencies in the public, commercial, or not‐for‐profit sectors.

## Ethics Statement

This article is a meta‐analysis of previously published studies and does not contain any studies with human participants or animals performed by any of the authors.

## Conflicts of Interest

The authors declare no conflicts of interest.

## Data Availability

Data used in this meta‐analysis were extracted from previously published studies listed in the references. Further details are available from the corresponding author upon request.
